# Tumor imaging and photothermal therapy in second near infrared window: A systematic review and meta-analysis

**DOI:** 10.3389/fonc.2022.987491

**Published:** 2022-09-08

**Authors:** Fuhan Fan, Ya Hou, Yating Zhang, Yong Zeng, Yi Zhang, Sanyin Zhang, Xianli Meng, Xiaobo Wang

**Affiliations:** ^1^ School of Pharmacy, Research Institute of Integrated TCM & Western Medicine Chengdu University of Traditional Chinese Medicine, Chengdu, China; ^2^ Ethnic Medicine Academic Heritage Innovation Research Center, Chengdu University of Traditional Chinese Medicine, Chengdu, China; ^3^ State Key Laboratory of Southwestern Chinese Medicine Resources, Innovative Institute of Chinese Medicine and Pharmacy, Chengdu University of Traditional Chinese Medicine, Chengdu, China

**Keywords:** NIR-II, tumor imaging, photothermal therapy, safety evaluation, meta-analysis

## Abstract

**Background:**

Second near-infrared window (NIR-II, 1000-1700 nm) technology for tumor imaging and photothermal therapy (PTT) is an innovative method for tumor diagnosis and treatment. The NIR-II probe can specifically identify tumor cells, and effectively convert light energy into heat energy under the irradiation of NIR laser, thus achieving the integration of non-invasive tumor diagnosis and treatment. In the present study, we conducted a systematic review and meta-analysis of preclinical investigations to corroborate the efficacy and safety of photothermal therapy.

**Methods:**

Relevant preclinical data were retrieved by searching PubMed, Web of Science, CNKI, WANFANG and VIP information databases. And the acquired data were analyzed by RevMan Version 5.3 software.

**Results:**

According to the inclusion criteria, forty-two articles relating to NIR-II tumor imaging and PTT were recruited for further in-depth analysis. The NIR-II photoacoustic and fluorescence imaging could quickly and accurately identify tumor in mice, manifesting higher signal intensity on tumor site than that of normal tissue. After PTT, the tumor volume of mice decreased miraculously [RR=8.49, 95%CI (4.64, 15.55), P<0.00001], and even disappeared completely [RR=7.01, 95%CI (3.04, 16.13), P<0.00001] with no potential risk of affecting the blood routine.

**Conclusions:**

PTT guided by NIR-II imaging can effectively diagnose the tumor lesion and eliminate it with the advantages of non-invasive and higher biosafety.

## Introduction

Tumor is a form of disease with excessive mortality globally with no effective and completely acceptable techniques for its cure and prognosis (1). Conventional non-invasive medical imaging devices, such as computed tomography (CT), magnetic resonance imaging (MRI), ultrasound (US) and positron emission tomography (PET) (2), have made some achievements in the detection of diversified tumors. However, the problems of low resolution, shallow imaging depth and inevitable radiation still force human beings to hunt for novel clinical imaging methods, which stimulate a new development in optical imaging. It is well worth noting that photons for biological imaging in visible light window will be scattered and absorbed by blood, fat and hemoglobin in organic tissues, making it difficult to photograph deep tissues (3). As a result, near-infrared (NIR) imaging and therapeutic methods with robust tissue penetration and small photons scattering have been swiftly developed in current years. Imaging guided *via* the first near infrared window (NIR-I, 700-900nm) and the NIR-II window is the predominant direction of NIR imaging (4). Even more thrillingly, the greater imaging depth and signal-to-noise ratio of the NIR-II window make it extra competitive in contrast with the NIR-I window (5). As a promising technology for tumor diagnosis, NIR-II fluorescence can label specific tissue structures at the molecular or cellular level, contributing to identify minimal residual tumors with high precision.

**Figure 1:**
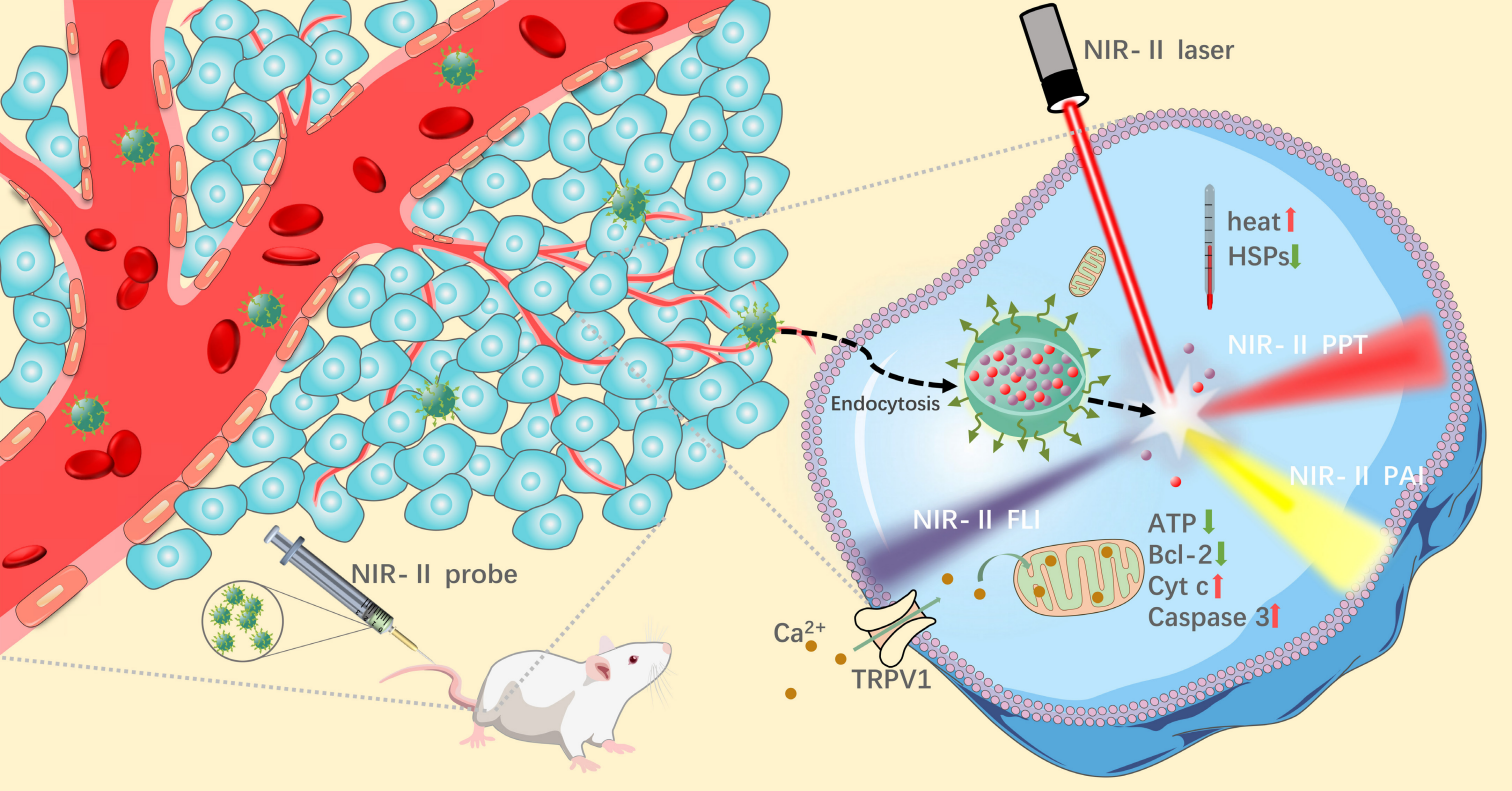
Schematic diagram of tumor imaging and PTT in the second near-infrared window.

In addition, some NIR probes can additionally be used as photothermal agents (PTA) to achieve photothermal therapy (PTT) of tumor cells under the irradiation of NIR laser (6). PTT is a cure approach that kills tumor cells by way of heating up the local tumor tissue. It has been reported that irreversible injury can be brought about to tumor cells when the temperature exceeds 43°C (7). The tumor site was accurately located by NIR-II imaging system *via* certain NIR laser irradiation to the tumor tissue. In principle, PTA can activate from ground state to excited state by absorbing photon energy under NIR laser irradiation, and then collide with surrounding molecules to generate kinetic energy and eventually convert into heat to kill tumor cells (8). After a persistent period of photothermal treatment, blood and major organs of tumor model mice were collected for blood routine and H&E staining analysis. It was suggested that a mass of the tumor cells went into apoptosis with no distinct difference in normal tissue and blood routine indexes between PTT-treated mice and normal mice (9, 10). Compared with traditional surgical resection for tumor treatment, NIR-II guided tumor imaging and PTT exhibit much less biological trauma and higher specificity (11), which was promising to be a novel approach for clinical treatment of tumors. The principles of tumor localization and photothermal treatment guided by NIR-II imaging are shown in **Figure 1**.

At present, NIR-II fluorescence imaging has made a great breakthrough in guiding clinical tumor resection. With the advantages of real-time imaging and wide field of view, it can provide dynamic feedback of tumor area information in the course of surgery. More accurately, numerous evidence demonstrated that NIR-II imaging can detect tumor signals that cannot be identified by CT imaging (12). Based on the achievements of NIR-II imaging in cancer surgery and the encouragement of preclinical data on photothermal treatment, we believe that NIR-II imaging guided PPT has a bright future in clinical cancer diagnosis and treatment. Due to the lack of relevant clinical data, we conducted a systematic review and meta-analysis of the preclinical data of PTT, providing more powerful evidence for its efficacy and safety.

## Methods

### Literature search

Two researchers (FF and YH) used PubMed, Web of Science, China National Knowledge Infrastructure (CNKI), WANFANG, and VIP Journal database to conduct a comprehensive search for publications up to January 10, 2022. The following is the literature search algorithm: (second near infrared OR NIR-II) AND (cancer OR tumor) AND (photothermal therapy OR PTT). In addition, there are no other relevant restrictions in the retrieval of literature search.

### Study selection

All the literatures were retrieved by the above programmed algorithm and were input into reference management software for unified management. Firstly, articles were preliminarily screened according to the title and abstract, and then the full context was read to cautiously consider whether to be subordinated to our study. Article selection criteria were carefully determined and strictly enforced during the study selection process. Studies were included when they conformed all of the following criteria: 1) Use of NIR-II imaging to locate tumors and cancer cells; 2) PTA related characterization; 3) Photothermal tests were carried out on mice using NIR-II imaging system, and data related to the efficacy and safety of PTT were obtained. Meanwhile, studies were excluded if they accorded with any of the following criteria:1) One of a range of reviews, case reports, conference articles, abstracts, social reviews, and clinical literature; 2) None of experimental study; 3) None of NIR-II imaging; 4) None of records associated to the characteristic of PTA; 5) None of information related to tumor imaging; 6) None of data related to PTT; 7) Duplicate publications.

### Data extraction and quality evaluation

The process of information extraction was mutually performed by two reviewers (FF and YZ). Pre-defined information extraction strategies were employed for effective extraction, and the differences between reviewers were resolved by consensus. The required data was extracted as comprehensively as possible from the included article, including text material and charts as sources of information. However, when solely charts were available in the article, the applicable information was thus obtained quantitatively on the charts using the line length tool in PowerPoint (13). The essential information collected were as follows: first author and publication time, basic information of the PTA, bioactivity of the probe, tumor imaging ability, *in vitro* tissue signals, *in vitro* and *in vivo* photothermal conversion parameters of the PTA, grouping and sample size, photothermal treatment conditions, and volume change of tumor after PTT.

The quality of the studies was evaluated through scoring 10 indicators in the preclinical research quality evaluation table: 1) Whether the articles were published after peer review; 2) Whether there is a description of temperature control; 3) Whether animal groups are randomly assigned; 4) Whether to follow the allocation hiding; 5) Whether the outcome indicators were evaluated by blind method; 6) Whether anesthetics with neuroprotective properties are used; 7) Whether animal models with comorbidities are used; 8) Whether to specify the sample size; 9) Whether there is a declaration of animal welfare regulations (10); Whether there is a conflict of interest related declaration. When there is a description in the article that conforms to the relevant indicators, it will get one point under this indicator. Otherwise, it will no longer get point. The ultimate rating of the paper was 0-10, with higher scores indicating more rigorous methodological design of research.

### Statistical analysis

RevMan version 5.3 software was used for data summary and analysis. Dichotomous variable method was used to characterize the efficacy of PTT in tumor treatment. The random-effects model and 95% confidence interval (CI) risk ratio (RR) were used to show the association. The continuous variable method was used to characterize the safety of PTT. Fixed effect model and mean difference (MD) of 95% CI were used to evaluate the changes of specific blood routine indexes. The I^2^ statistical test was used to test the heterogeneity of individual studies. When I^2^>50% of the time, we thought the study had significant heterogeneity. While the probability value less than 0.05 was considered statistically significant.

## Results

### Literature search

**Figure 2:**
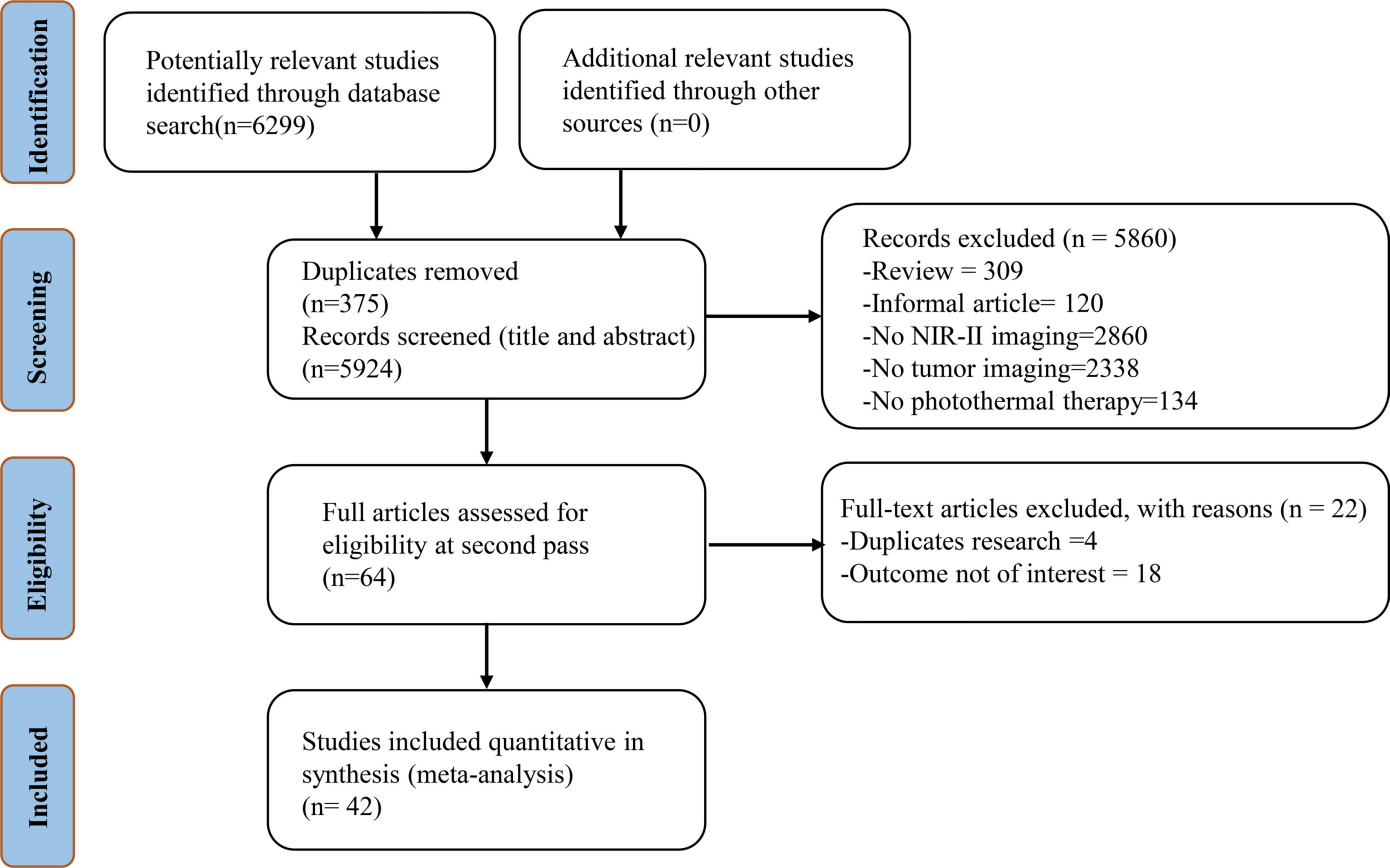
Flow chart of study selection.

A total of 6299 related studies were obtained from the target database by the predefined search algorithm. And 5924 articles were retrieved by excluding duplicate articles. By reading the title and abstract of the articles, 5860 articles that did not conform the requirements were initially excluded, including 309 non-experimental articles, 120 informal articles (news reports and comments), 2860 articles that did not involve NIR-II imaging, 2338 articles that did not contain tumor imaging, and 134 articles that did not use PPT. The remaining 64 studies were then screened and evaluated through full-text reading, and 22 papers were further excluded. Finally, 42 related studies were included in the meta-analysis (7–10, 14–51). The retrieval and selection process of the article is shown in **Figure 2**.

### Study characteristics

Different kinds of probes have widespread differences in the outcomes of PTT. We sorted out the essential traits of the probes concerned in the studies (**Table 1**), including excitation wavelength, quantum yield (QY) and extinction coefficient (ϵ), et al. Due to the innovativeness of NIR-II image-guided PTT utilized to tumors treatment, all the included studies were published in latest years (2018 to 2021) and were preclinical researches. The type of probe imaging comprised NIR-II fluorescence imaging (21 researches), NIR-II photoacoustic imaging (6 researches), and NIR-II fluorescence photoacoustic dual-mode imaging (15 researches). The probes involved in each study were reported for the first time and were no longer reproducible with each other.

**Table 1:** The basic information of NIR-II probe included in the study.

Study	Names	Shape	Size (nm)	Type	A_max_ (nm)	E_max_ (nm)	QY (%)	ϵ (L/g.cm)	Cells Administration	Viability
Zhang 2021 (7)	SH-PEG-FA	Flake	37.6×1.5	PAI	NR	785, 1064	NR	32.58 (808 nm), 27.85 (1064nm)	HeLa (0~150 μg/ml, 24 h) 4T1 (0~150 μg/ml, 24 h)	76%, 82%
Dai 2021 (8)	Lip (DPQ+2DG)	Sphere	100	FI	1015	1300	0.02	26.7 (1064nm)	4T1 (0~625 μg/mL, 24 h)	75.03%
Li 2020 (9)	FS-GdNDs	NR	9.3 ± 0.5	FI	NR	NR	NR	NR	4T1 (0~5 μg/ml, NR)	85%
Wang 2019 (10)	N-B-GQDs	NR	4.7	FI	NR	1000	1.00	NR	4T1 (0~500 μg/ml, 72 h)	94%
Zheng 2021 (14)	HSC-2	Jujube	142.2	PAI/FI	NR	957	0.41	2.01	4T1 (0-400 μg/ml, 24 h)	85%
Zheng 2020 (15)	Ag_2_S-GOx@BHS Nys	Sphere	91.87	FI/PAI	NR	NR	NR	NR	4T1 (0-1000 μg/ml, 24 h)	75%
Zheng 2020 (16)	Bi_2_S_3_-Ag2S-DATS@BSA-N3 Nys	Sphere	8.8 ± 1.2	FI/PAI	900	1000	NR	NR	Hep2 (0-600 μg/ml, 12 h)	98.23%
Zhao 2020 (17)	QT-RGD	Sphere	34.7 ± 0.6	PAI	1042, 841	1068	0.12	NR	4T1 (0~20 μM, 24 h) NIH/3T3 (0~20 μM, 24 h)	>85%
Zhang 2020 (18)	TSSI NPs	Sphere	40	FI/PAI	664	NR	NR	27.2 (785 nm), 3.1 (1064 nm)	4T1 (0-50 μg/ml, 24 h)	>96%
Zhang 2020 (19)	PSQPNs-DBCO	Sphere	35 ~ 85	FI	1064	1290	NR	NR	NR	NR
Zhang 2019 (20)	SPNs3	Sphere	43 ~ 57	PAI	899, 1000, 1300	1280	NR	2.54 (1064 nm)	4T1 (0~50 μg/ml, 48 h)	94%
Yin 2019 (21)	OSPA	Sphere	69.7 ± 2.3	PAI	1000	NR	NR	18 (980 nm)	4T1 (0~0.3 mg/ml, NR)	>90%
Yang 2020 (22)	L1057 NPs	Sphere	51	FI	470, 937	1057	1.25	NR	3T3 (0~50 μg/ml, 24 h) LO2 (0~50 μg/ml, 24 h) MEF (0~50 μg/ml, 24 h)	>85%
Xie 2021 (23)	PFG MPNs	Sphere	80	FI/PAI	826	1063	NR	NR	B16F10 (0~1.250 mg/ml, 6 h)	>90%
Wang 2019 (24)	PEG-CSS@PB	Sphere	164	FI	NR	1525	NR	NR	HeLa (0~600 μg/ml, 24 h)	≈100%
Sun 2021 (25)	TTQ-MnCO NPs	Sphere	120	FI	NR	1115	NR	NR	MCF-7 (0~0.1 mg/ml, 24 h)	95%
Sun 2020 (26)	MNPH2	NR	NR	FI/PAI	NR	609, 1006	0.20	NR	Hep-2 (0~800 μg/mL, NR)	≈100%
Shi 2018 (27)	Nano-PT	NR	NR	FI	790	NR	NR	130.3 (750 nm)	HCT116 (0~20 μM, NR)	96%
Li 2021 (28)	ETTC NPs	Sphere	59.3	FI	750	NR	3.00	NR	A549 (0~50 μg/mL, 24 h)	90%
Li 2019 (29)	NaLuF4 NRs@PDA	Slavate	20 × 130	FI	NR	1525	1.37	5.09×10^5^ (M^-1^cm^-1^)	HeLa (0~1000 μg/ml, 24 h)	87%
Li 2019 (30)	PF	NR	90	FI	1026	1064	0.30	NR	4T1 (0~2.5 μg/ml, 24 h) HepG2 (0~2.5 μg/ml, 24 h)	85%, 91%
Li 2021 (31)	Pry-Ps@CP-PEG	Sphere	80	FI	900	1140	2.20	NR	4T1 (0~1 mg/m, NR)	≈90%
Li 2019 (32)	PFTDPP-SNAP NPs	NR	NR	PAI	NR	1060	1.8	NR	MCF-7 (0~1 mg/ml, NR)	>90%
Li 2021 (33)	BBTD-BET	Sphere	29 ± 3	FI/PAI	780	1094	0.004	NR	MCF-7 (0~800 μg/ml, 24 h)	>90%
Hu 2019 (34)	PFTQ-PEG-Gd NPs	Sphere	105	FI/PAI	NR	1056	0.38	10.36 (1064 nm)	NIH-3T3 (0~120 μg/ml, 24 h) 4T1 (0~120 μg/ml, 24 h) Hela (0~120 μg/ml, 24 h)	88%, 91%, 90%
Dai 2021 (35)	Lips (PTQ/GA/AIPH)	Sphere	85	FI/PAI	840	1300	NR	11.8	MDA-MB-23 (0~50 μg/ml, NR) NIH-3T3 (0~50 μg/ml, NR)	85.64%, 90%
Chen 2021 (36)	P1 NPs	Sphere	96	FI	705	1257	0.10	NR	4T1 (0~40 ug/ml, 48 h)	92%
Jia 2021 (37)	HSC	Sphere	200	FI/PAI	NR	955	NR	NR	4T1 (0~400 μg/ml, 12 h)	90%
Qian 2021 (38)	HQS-Cy@P	Sphere	53	FI	986	1050	NR	NR	HUAEC (0~20 μg/ml, NR)	85%
Guan 2021 (39)	SPIO@NC	NR	60	FI	NR	NR	NR	3.35×10^4^ (M^-1^cm^-1^)	NR	NR
Xia 2020 (40)	DPP-BDT NPs	Sphere	90	FI/PAI	625	980	NR	3.05×10^4^ (M^-1^cm^-1^)	NIH-3T3 (0~25 μg/ml, 24 h)	80%
Xu 2020 (41)	P (DPP-BT/DOX) NPs	Sphere	100	FI/PAI	730	1076	0.42	NR	NIH3T3 (0~50 μg/ml, NR)	86%
Zhu 2020 (42)	PCTA-BMA-OXA (PBOXA)	Sphere	75.12 ± 2.19	FI	NR	1040	NR	NR	NR	NR
Yao 2020 (43)	SQ1	NR	NR	FI/PAI	940	980	1.70	26.16 (930 nm)	MDA-MB231 (0~512 μg/ml, 24 h)	80%
Wang 2019 (44)	NPs-DB	Sphere	120	FI	837, 930	1082	NR	NR	4T1 (0~1 mg/mL, 48h)	85%
Meng 2018 (45)	IR1048-MZ	NR	NR	FI/PAI	980	1046	0.60	NR	A549 (0~100 μg/ml, 24 h)	56%
Zheng 2021 (46)	Pt⁃Cu@PLL@HA	Areatus	56.2	PAI	NR	NR	NR	NR	4T1 (0~400 μg/ml, 24 h)	95%
Huang 2020 (47)	BDT-TTQ NPs	Sphere	100	FI	1064	1300	NR	NR	HeLa (0~0.75mg/ml, 48 h)	80%
Zhou 2020 (48)	H4-PEG-PT	NR	(180.0 ± 13) × (48 ± 15)	FI	800	1050	2.01	3.4	143B (0~32 μM, 48 h) L929 (0~32 μM, 48 h)	87%, 96%
Yang 2017 (49)	Ag_2_S NDs	NR	32.8	FI	NR	1060	1.32	30	4T1 (0~0.5 mM, 24 h)	~100%
Feng e 2019 (50)	IR-820	NR	NR	FI	NR	NR	2.521	NR	NR	NR
Zhao 2019 (51)	CPCC-Ag_2_SQDs	Sphere	3.44	FI/PAI	NR	1099	3.78	NR	Hela (0~400 g/ml, 24 h)	80%

A_max_ (nm), maximum absorption wavelength; E_max_ (nm), maximum emission wavelength; ϵ, extinction coefficient; NR, not report; QY, quantum yield.

The emission wavelength, QY and ϵ of the probe are considered as vital elements affecting the fluorescence brightness of the probe. QY refers to the fluorescence conversion rate of fluorescent probe after absorption of photons (52). Included in the literature studied in this paper, the highest QY of the probe is 3.78% (51), while the lowest is 0.004% merely (30). Almost all of the probes involved in the studies had the maximum emission peak in the NIR-II region, despite of a few of the probes (37, 40, 43) with no maximum emission peak in the NIR-II region, but still had a large amount of fluorescence emission in this region. The ϵ of the probe at the maximum emission wavelength was further measured, and the maximum and minimum ϵ of the articles included in this paper were 130.3 and 2.01 L/cm·g (14, 24), respectively. In addition, the cell viability of probes with different concentrations and related cells cultured for a certain period of time was calculated as the important biosafety evaluation index of probes. The cell survival rate of the probes used in this study was above 80%, and the influence of some probes on cell activity even could be ignored (16, 24, 26, 46, 49).

### Tumor imaging capability of NIR-II

The probe is the basis of PTT for high-precision imaging of tumors in the NIR-II imaging system. In this regard, we thus sorted out the imaging information of different probes in **Table 2**. The capacity of the probe to image tumor can be expressed by the time of the maximum signal of NIR-II fluorescence imaging or photoacoustic imaging, and the ratio of the maximum signal to the initial signal. The shorter time of the maximum signal intensity indicated the stronger selectivity of the probe to the tumor. And the larger ratio of the maximum signal to the initial signal manifested the potential of imaging to a certain extent. In this paper, the maximum signal time of the fluorescent probe is mostly 12 h and 24 h, the shortest and longest time are 3 h (49) and 5 d (44) respectively. Noteworthily, the ratio of the maximum signal to the initial signal can be enhanced up to 15 times (40). Most of the time of the maximum signal of the photoacoustic probe is between 6-24 h with the ratio of the maximum signal to the initial signal up to about 13 times (17, 47).

**Table 2:** The characteristic profiles of probes for tumor imaging.

Study	Animal	Probe dose	PAI of tumor	FI of tumor	Ac	*In vitro* tissue signal
Zhang 2021 (7)	NR	NR	NR	NR	NR	NR
Dai 2021 (8)	NR	800μg/ml, iv.	NR	24 h, 6.7 times	0.402 mm	Liver>spleen>tumor, 48 h
Li 2020 (9)	Female BALB/c nude mice	NR	NR	12 h, NR	NR	Liver>spleen>tumor, 24 h
Wang 2019 (10)	Nude mice	1mg/ml 200μl, iv.	NR	NR	130 μm	Liver>kidney>spleen, 24 h
Zheng 2021 (14)	NR	20 mg/kg, iv.	12 h, 10 times	12 h, 8.34 times	NR	Liver>tumor>spleen, 24 h
Zheng 2020 (15)	BALB/c nude mice, 15-17 g	20 mg/kg, iv.	8 h, 7.5 times	8 h, 9.4 times	NR	Liver>tumor>spleen, 24 h
Zheng 2020 (16)	Female BALB/c nude mice, 6~8 w, 18~20 g	15 mg/kg, iv.	6 h, NR	6 h, NR	NR	Liver>tumor>spleen, 24 h
Zhao 2020 (17)	BALB/c mice	100 μM 200 μl, iv.	8 h, 13.8 times	5 h, 3.6 times	0.43 mm	Tumor>kidney>liver, 24 h
Zhang 2020 (18)	BALB/c nude mice	NR	NR	12 h NR	NR	Liver>spleen>tumor, 24 h
Zhang 2020 (19)	BALB/c mice, 6 w	2 mg/ml 100 μl, iv.	NR	NR	NR	NR
Zhang 2019 (20)	NR	1 mg/ml 100 μl, iv.	10 h, 2 times	NR	NR	NR
Yin 2019 (21)	NR	NR	6 h, 8 times	NR	NR	Liver,24 h
Yang 2020 (22)	NR	500 μg/kg 80 μl, iv.	48 h, 4.3 times	NR	1.9 μm	Liver>tumor>spleen, 48 h
Xie 2021 (23)	C57BL/6 mice, 6-8 w, 18 ± 2 g	NR	24 h, 4.1 times	24 h, 6.7 times	NR	Liver>spleen, 36 h
Wang 2019 (24)	Female BALB/c mice	0.5 mg/ml, iv.	NR	NR	NR	NR
Sun 2021 (25)	NR	2 mg/ml 150 μl, iv.	NR	24 h, 8 times	0.35 mm	Spleen>liver>tumor, 26 h
Sun 2020 (26)	BALB/c mice, 6~8 w, 18~20 g	800 μg/ml 200 μl, iv.	8 h, 2.9 times	8 h, 2.2 times	NR	Liver>kidney>tumor, 24 h
Shi 2018 (27)	NR	NR	NR	2 h, NR	NR	NR
Li 2021 (28)	Female BALB/c mice,5~6 w	1 mg/ml 200 μl, iv.	12 h, 6.8 times	12 h	NR	Liver>spleen>tumor, 12 h
Li 2019 (29)	NR	0.2 mg/ml 0.2 ml, iv.	NR	NR	45 μm	Liver>tumor, 60 h
Li 2019 (30)	BALB/c mice	NR	NR	12 h, NR	NR	Liver>lung>kidney, 24 h
Li 2021 (31)	NR	4 mg/ml 200 μl, iv.	NR	24 h, NR	NR	NR
Li 2019 (32)	Female BALB/c mice, 4~5 w	1 mg/ml, iv.	24 h, times	24 h, NR	NR	Liver>spleen>tumor, 24 h
Li 2021 (33)	NR	10 mg/ml 200 μl, iv.	NR	12 h, NR	0.675 mm	Liver>tumor>spleen, 24 h
Hu 2019 (34)	NR	1mg/ml 150μl, iv.	24 h, 4.5 times	24 h, NR	NR	Spleen>liver>tumor, 24 h
Dai 2021 (35)	NR	NR	24 h, 9.3 times	24 h, 4.2 times	0.42 mm	Spleen>liver>tumor, 36 h
Chen 2021 (36)	NR	2mg/ml 100ul, iv.	NR	24 h, 2.8 times	0.4 mm	Tumor>liver>spleen, 48 h
Jia 2021 (37)	Female Nude mice, 20 g	20mg/kg, iv.	NR	12 h, NR	NR	NR
Qian 2021 (38)	Female BALB/c mice, 5 w	8mg/kg 150μl, iv.	12 h	8 h, NR	0.705 mm	Liver, 8 h
Guan 2021 (39)	Female BALB/c mice, 6 w	20μg/ml 200μl, iv.	NR	24 h, NR	NR	Tumor>lung>liver, 48 h
Xia 2020 (40)	NR	2mg/ml 100μl, iv.	NR	20 h, 2.8 times	NR	NR
Xu 2020 (41)	NR	NR	20 h, 15 times	24 h, 9.4 times	NR	Liver>spleen>tumor, 72 h
Zhu 2020 (42)	Female BALB/c mice, 18~20 g	NR	24 h, 10.5 times	48 h, NR	NR	Liver>spleen>tumor, 48 h
Yao 2020 (43)	NR	5mg/kg, iv.	NR	12 h, NR	NR	NR
Wang 2019 (44)	BALB/c mice	0.25mg/ml 50μl, iv.	12 h, NR	5 d, NR	NR	Liver>kidney>spleen, 21 h
Meng 2018 (45)	Female BALB/C mice, 6~8 w, 15~20 g	40μg/ml 200μl, iv.	NR	14 h, NR	NR	Tumor>lung>liver, 14 h
Zheng 2021 (46)	Female BALB/c, 6~8 w, 17~19 g	15mg/kg, iv.	14 h, NR	24 h, 6 times	NR	NR
Huang 2020 (47)	BALB/c mice	2mg/ml 100μl, iv.	12 h, 13.4 times	12 h, 3.8 times	NR	NR
Zhou 2020 (48)	Female BALB/c nude mice, 6 w	400μg/ml 200μl, iv.	NR	12 h, NR	NR	Liver, 48h
Yang 2017 (49)	BALB/c mice	50.0umol/kg iv.	NR	4 h, 3 times	NR	Tumor>liver>kidney, 24 h
Feng 2019 (50)	Female ICR mice, 6~7 w; Male BALB/c mice, 6~7 w	2mg/ml 100μl, iv.	24 h, 2.4 times	48 h, NR	2.496 μm	Liver>kidney, 48 h
Zhao 2019 (51)	NR	150μg/ml 100μl, iv.	NR	NR	NR	NR

Ac, the vessel width that the probe can present in the second near-infrared window; NR, not report.

In general, intravenously injected probes can image biological blood vessels to varying degrees. The smaller the visualized vessels by NIR-II imaging system indicates the higher accuracy of the probe to some extent. Most of the probes included in this meta-analysis were able to image mice blood vessels with a diameter of about 0.4 mm (8, 17, 35, 36), while some even reached ~1.9 μm with a high-magnification imaging system (22). In addition, the blood vessels interacting with tumor tissues can be clearly observed by high-power imaging systems (29), which is of great meritorious for monitoring tumor changes during PTT. In most studies, signal intensities in tumor blocks, liver and spleen were highest at 12 h, 24 h, and 48 h (8, 9, 14–16, 18, 22, 28, 32–34, 36, 42), which then began to decline continuously. This indicates that the involved probes have higher selectivity for tumor and specific tissues. And the accumulated probe is excreted mainly through the hepatobiliary system.

### Effect of PTT guided by NIR-II imaging

The effect of periodic PTT is ultimately reflected in the actual change of tumor volume. In order to further analyze the effect of photothermal treatment, we collated the photothermal information of different PTA and the detailed changes condition of tumor *in vivo* (**Table 3**). Among the included studies, there were 17 items whose PTA photothermal conversion efficiency was greater than 40%, 20 items whose probe photothermal conversion efficiency was lower than 40%, and 6 items had no relevant records. PTT typically makes use of 1.0 w/cm^2^ at 1064 nm (8, 14, 19, 20, 21, 25, 35–37, 46, 47) and 1.0 W/cm^2^ at 808 nm (15–17, 23, 24, 26, 28, 31, 32, 34, 39, 42) NIR light sources to irradiate the tumor site. And a few studies also choose other light sources according to the properties of PTA [e.g., 0.1 W/cm^2^ at 980 nm (45), and 1 W/cm^2^ at 915 nm (38)]. The PTA with excellent photothermal conversion efficiency can make the tumor heat up rapidly in a short time after feeling the NIR laser irradiation. The maximum temperature of PTA was higher than 43 °C in 31 studies, which could theoretically cause irreversible injury to tumors. Under the continuous photothermal treatment with a general period of 14 to 30 days, the tumor necrosis was gradually accompanied by a marked reduction in size.

**Table 3:** The characteristic profiles of PTA for photothermal treatment.

Study	*In vitro* photothermal capacity	η (%)	Administration	Conditions of PTT	V_0_ (mm^3^)	Times (h)	Days (d)	△T (°C)	T_max_ (°C)	Effect
Zhang 2021 (7)	100 μg/ml, 1064 nm, 6 min△T=28°C	45.7	NR	NR	NR	NR	21	20.2	50.2	–
Dai 2021 (8)	NR, 1064 nm, 5 min,△T=45.4°C	40.92	800 μg/ml	1064 nm, 1.0 W/cm^2^, 10 min	NR	24	15	21.5	50	↑
Li 2020 (9)	5 μg/ml, 808 nm, 10 min,△T=15.8°C	43.99	3.5 mg/kg	808 nm, 0.96 W/cm^2^, 10 min	NR	12	16	18.1	49.1	–
Wang 2019 (10)	200 μg/ml, 808 nm, 5 min,△T=26.6°C	32.32	1 mg/mL 200 μl	808 nm, 1.5 W/cm^2^, 5 min	NR	NR	14	NR	NR	↓
Zheng 2021 (14)	400 μg/ml, 1064 nm, 5min, △T=34°C	41.41	20 mg/kg	1064 nm, 1.0 W/cm^2,^5 min	80	12	10	17.5	NR	–
Zheng 2020 (15)	NR	NR	20 mg/kg	808 nm, 1.0 W/cm^2^, 5 min	100	8	15	22.5	58.5	↓
Zheng 2020 (16)	600 μg/ml, 808 nm, 5 min, △T=36.9°C	31.60	15 mg/kg	808 nm, 1.0 W/cm^2^, 5 min	100	6	20	17.5	NR	–
Zhao 2020 (17)	NR	36.50	100 μM 200 μl	808 nm, 1.0 W/cm^2^, 10 min	20	4	21	21.7	NR	–
Zhang 2020 (18)	100×10^-6^ m, 660 nm, 5 min,△T=28°C	46	NR	660 nm, 0.3 W/cm^2^, 5 min	NR	12	15	17.5	54.8	–
Zhang 2020 (19)	125 μg/ml, 1064 nm, 5min,△T=50°C	33.4	2 mg/ml 100 μl	1064 nm, 1.0 W/cm^2^, 5 min	NR	24	15	NR	NR	↑
Zhang 2019 (20)	25 μg/ml, 1064 nm, 400 s,△T=44.5°C	60	1 mg/ml 100 μl	1064 nm, 1.0W/cm^2^, 5 min	NR	10	15	45	75.2	↑
Yin 2019 (21)	0.8 mg/ml, 1064 nm, 6 min,△T=59.5°C	30.53	NR	1064 nm, 1.0 W/cm^2^, 5 min	100	6	14	26.2	58.2	–
Yang 2020 (22)	100 μg/ml, 980 nm, 500 s,△T=27.3°C	38	500 μg/kg 80 μl	980 nm, 0.72W/cm^2^, 10 min	100	1	14	24	58	↓
Xie 2021 (23)	0.25 mg/ml, 808 nm, 10 min,△T=73°C	75.60	NR	808 nm, 1.0 W/cm^2^, 4 min	80	24	14	18.5	50	↑
Wang 2019 (24)	600 μg/ml,808 nm, 10 min,△T=23°C	50.50	0.5 mg/ml	808 nm, 1.0 W/cm^2^, 10 min	90	NR	13	NR	42	↓
Sun 2021 (25)	0.10 mg/ml, 1064 nm, 5min,△T=44.8°C	44.43	2 mg/ml 150 μl	1064 nm, 1.0 W/cm^2^, 5 min	NR	NR	15	23.5	56	↓
Sun 2020 (26)	200 μg/ml, 808 nm, 5 min,△T=30.8°C	NR	800 μg/ml 200 μl	808 nm, 1.0 W/cm^2^, 5 min	100	8	16	25	59.2	–
Shi 2018 (27)	NR,10 min,△T=32°C	27.80	NR	785 nm, 1.66 W/cm^2^, 5 min	NR	2	15	30.9	60.9	–
Li 2021 (28)	50 μg/ml, 808 nm, 8 min,△T=58°C	52.80	1 mg/ml 200 μl	808 nm, 1.0 W/cm^2^, 10 min	100	12	14	27	NR	–
Li 2019 (29)	0.2 mg/ml, 808 nm, 10 min,△T=34°C	40.18	0.2 mg/ml 0.2 ml	808 nm, 10 min	NR	NR	12	NR	NR	–
Li 2019 (30)	25 μg/ml, 808m, 5 min,△T=29.8°C	42.30	NR	808 nm, 0.65 W/cm^2^, 10 min	150	1	21	20.3	51.8	–
Li 2021 (31)	1 mg/ml, 808 nm, 5 min,△T=47°C	43.70	4 mg/mL 200 μl	808 nm, 1.0 W/cm^2^, 5 min	80	24	15	31.5	NR	–
Li 2019 (32)	2 mg/ml, 808 nm, 10 min,△T=60°C	48	1 mg/ml	808 nm, 1.0 W/cm^2^, 10 min	NR	24	21	28	60	–
Li 2021 (33)	2 mg/ml, 808 nm, 5 min,△T=45.7°C	39.42	10 mg/ml 200 μl	808 nm, 0.3 W/cm^2^, 10 min	100	12	14	21	56	–
Hu 2019 (34)	500 μg/ml, 808 nm, 10 min,△T=41°C	26	1 mg/mL 150 μl	808 nm, 1.0 W/cm^2^, 10 min	80	24	16	25	58.5	↑
Dai 2021 (35)	80 μg/ml, 1064 nm, 5 min,△T=45.8°C	39.24	NR	1064 nm, 1.0 W/cm^2^, 10 min	NR	24	14	18	NR	↑
Chen 2021 (36)	40 μg/ml, 1064 nm, 400 s,△T=25°C	55.10	2 mg/mL 100 ul	1064 nm, 1.0 W/cm^2^, 5 min	100	24	15	20.7	53.2	↑
Jia 2021 (37)	400 μg/ml, 1064 nm, 5 min,△T=34°C	NR	20 mg/kg	1064 nm, 1.0 W/cm^2^, 5 min	100	12	10	NR	NR	–
Qian 2021 (38)	20 μg/ml, 915 nm, 5 min,△T=24.2°C	35.50	8 mg/kg 150 μl	915 nm, 1.0 W/cm^2^, 10 min	100	8	15	21.7	48	–
Guan 2021 (39)	20 μg/ml, 808 nm, 5 min,△T=30°C	12.91	20 μg/ml 200 μl	808 nm, 1.0 W/cm^2^, 5 min	500	24	14	16.9	50.1	–
Xia 2020 (40)	100 μg/ml, 660 nm, 10 min,△T=26°C	23	2 mg/ml 100 μl	660 nm, 0.3 W/cm^2^, 10 min	100	20	14	NR	50	↑
Xu 2020 (41)	100 μg/ml, 730 nm, 10 min,△T=45°C	50.00	NR	730 nm, 1.0 W/cm^2^, 10 min	NR	24	14	NR	54	↑
Zhu 2020 (42)	80 μM, 808 nm, 10 min,△T=62°C	NR	NR	808 nm, 1.0 W/cm^2^, 10 min	50	24	16	18.2	53.7	↑
Yao 2020 (43)	1 mg/ml, 915 nm, 5 min,△T=41.2°C	25.60	5 mg/kg	915 nm, 0.5 W/cm^2^, 5 min	NR	12	14	25.9	59.1	–
Wang 2019 (44)	0.1 mg/ml, 915 nm, 6 min,△T=40°C	58.20	0.25 mg/mL 50 μl	915 nm, 0.5 W/cm^2^, 10 min	120	6	15	48	68	↑
Meng 2018 (45)	100 μg/ml, 808 nm, 5 min,△T=37°C	20.20	40 μg/mL 200 μl	980 nm, 0.1W/cm^2^, 2 min	100	14	30	30	57	–
Zheng 2021 (46)	400 μg/ml, 1064 nm, 5 min,△T=36°C	NR	15 mg/kg	1064 nm, 1.0 W/cm^2^, 5 min	75	12	12	17.89	51.9	–
Huang 2020 (47)	1 mg/ml, 1064 nm, 5 min,△T=35°C	NR	2 mg/mL 100 μl	1064 nm, 1.0 W/cm^2^, 5 min	100	12	15	24.5	52	↑
Zhou 2020 (48)	64 uM, 808 nm, 12 min,△T=30.5°C	18	400 μg/ml 200 μl	808 nm, 1.5 W/cm^2^, 8 min	800	12	14	34.8	67.8	↓
Yang 2017 (49)	1.0 mM, 808 nm, 5 min,△T=35°C	35.0	50.0 umol/kg	785 nm, 1.5 W/cm^2^, 5 min	60	24	30	19.0	NR	–
Feng 2019 (50)	500 μg/ml, 793 nm, 3 min,△T=70.5°C	32.74	2 mg/ml 100 μl	793 nm, 2.0 W/cm^2^, 10 min	120	48	16	24.7	55.4	↑
Zhao 2019 (51)	600 μg/ml, 808 nm, 6 min,△T=45°C	21	150 μg/ml 100 μl	808 nm, 2.5 W/cm^2^, 10min	100	NR	18	61.2	61.2	↑

↑, tumor volume increases; ↓, tumor reduced in size but did not disappear completely; -, complete tumor ablation; η, photothermal conversion efficiency; △T, tumor temperature changes after laser irradiation; Days, treatment cycle; NR, not report; Times, the time at which the NIR laser irradiation began after the probe was given; T_max_, maximum temperature of tumor after laser irradiation; V_0_, the initial volume of tumor.

**Figure 3:**
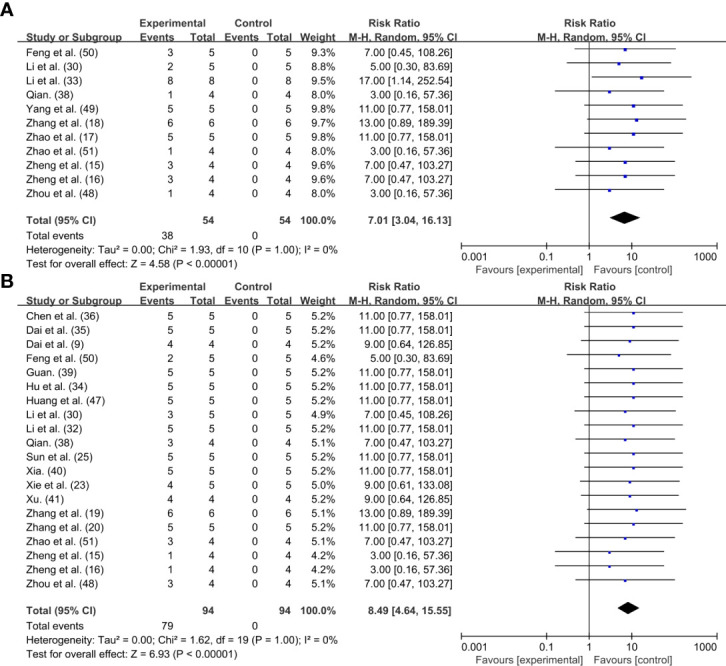
Forest plot for PTT effect: **(A)** Tumor disappeared completely; **(B)** Tumor shrank but did not completely disappear.

Finally, we used the forest plot to analyze the curative effect of PTT based on the proportion of tumor cured cases to the total sample size. The total sample size of individual studies and the number of cases with different efficacy were further calculated by reviewed the included studies and the supplementary literatures. Of the 42 studies, 11 items confirmed that tumors could be completely ablated by photothermal treatment (15–18, 30, 33, 38, 48–51). And 20 items showed conspicuous reduction in tumor size but not complete elimination (8, 15, 16, 19, 20, 23, 25, 30, 32, 34–36, 38–41, 47, 48, 50, 51). Compared with the control group, tumors treated with periodic PTT could eventually be completely ablated with some degree of crusting [n=108, RR=7.01, 95%CI (3.04, 16.13), P<0.00001; Heterogeneity: Tau^2 =^ 0.00, Chi^2 =^ 1.93, df=10 (P=1.00), I^2 =^ 0%] (**Figure 3A**). At the same time, though most of the PTA did not completely ablate the tumor with the aid of PTT, but the tumor volume exhibited a prominent trend of reduction comparing with initial size [n=188, RR=8.49, 95%CI (4.64, 15.55), P<0.00001; Heterogeneity: Tau^2 =^ 0.00, Chi^2 =^ 1.62, df=19 (P=1.00), I^2 =^ 0%] (**Figure 3B**).

### Safety evaluation of PTT

Blood routine test can reflect the pathological changes of the body by observing the changes in the number and distribution of blood cells. In this study, 11 researches measured the blood routine of animals after PTT, and the main indexes were WBC, RBC, HGB, MCV, MCH and MCHC (7, 17–20, 22, 27, 28, 33, 34, 50). Unfortunately, we solely obtained detailed data from four researches for meta-analysis (7, 18, 33, 50), two of which were tumor mice (7, 18) and two were healthy mice (33, 50). Through data analysis, it was found that the blood routine indexes except WBC were mostly within the reference range, indicating that PTT has high security for tumor treatment.

**Figure 4:**
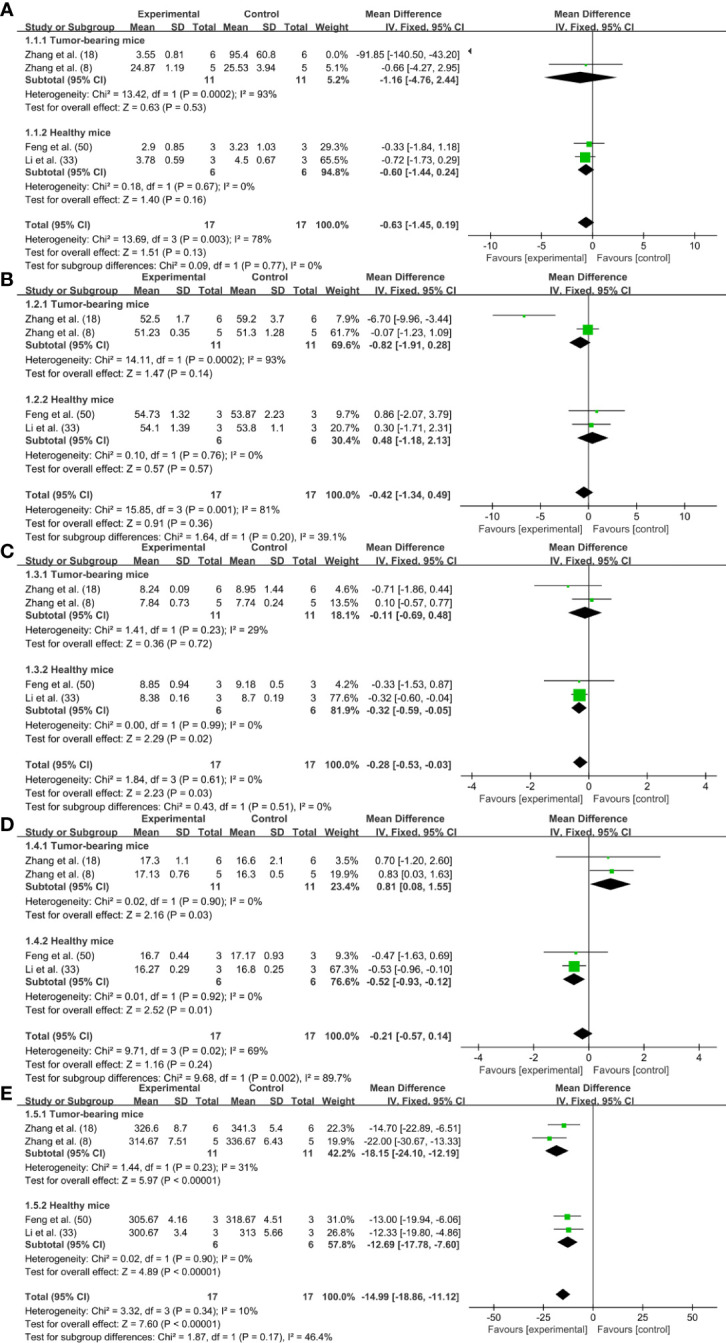
Forrest plots for blood routine: **(A)** WBC; **(B)** RBC; **(C)** MCV; **(D)** MCH; **(E)** MCHC.

In order to analyze the particular changes of relevant indicators, we conducted a forest plot on the data of four researches. The changes of WBC [n=34, MD=-0.63, 95%CI (-1.45, 0.19), P=0.13; Heterogeneity: Chi^2 =^ 13.69, df=3 (P=0.003), I^2 =^ 78%] (**Figure 4A**) and MCV [n=34, MD=-0.42, 95%CI (-1.34, 0.49), P=0.36; Heterogeneity: Chi^2 =^ 15.85, df=3 (P=0.001), I^2 =^ 81%] levels in mice after periodic PTT had no effect (**Figure 4B**). The changes of RBC level in tumor mice showed no distinction from the control group, while reduced slightly in healthy mice [n=34, MD=-0.28, 95%CI (-0.53, -0.03), P=0.03; Heterogeneity: Chi^2 =^ 1.84, df=3 (P=0.61), I^2 =^ 0%] (**Figure 4C**). Although the trend of MCH level was different in tumor mice and healthy mice, the final aggregate effect showed no difference with the control group [n=34, MD=-0.21, 95%CI (-0.57, 0.14), P=0.24; Heterogeneity: Chi^2 =^ 9.71, df=3 (P=0.02), I^2 =^ 69%] (**Figure 4D**). MCHC levels were down regulated in both tumor and healthy mice after periodic PTT [n=34, MD=-14.99, 95%CI (-18.86, -11.12), P<0.00001; Heterogeneity: Chi^2 =^ 3.32, df=3 (P=0.34), I^2 =^ 10%] (**Figure 4E**).

### Quality score

The 42 studies were evaluated according to the study quality evaluation index of preclinical research, and the average score of 6.52 is shown in **Table 4**. The overall score of the researches ranged 3~9, among which 23 studies scored higher than 7 (8–10, 15, 16, 19, 21, 22, 24–26, 28, 29, 32–34, 38, 44–46, 48–50), and 19 studies scored between 3~6 (7, 14, 17, 18, 20, 23, 27, 30, 31, 35–37, 39–43, 47, 51). The main factors causing the subtraction of the research were lack of description of temperature, types of narcotic drugs, and randomization information.

**Table 4:** Quality assessment of the experiments included in the studies.

Study	1	2	3	4	5	6	7	8	9	10	Total
Zhang 2021 (7)	+	NR	NR	+	+	NR	+	+	NR	+	6
Dai 2021 (8)	+	NR	+	+	+	NR	+	+	+	+	8
Li 2020 (9)	+	NR	+	+	+	+	+	+	+	+	9
Wang 2019 (10)	+	NR	+	+	+	+	+	+	+	NR	8
Zheng 2021 (14)	+	NR	NR	+	+	NR	+	+	NR	+	6
Zheng 2020 (15)	+	NR	NR	+	+	NR	+	+	+	+	7
Zheng 2020 (16)	+	NR	NR	+	+	NR	+	+	+	+	7
Zhao 2020 (17)	+	NR	NR	+	+	NR	+	+	NR	+	6
Zhang 2020 (18)	+	NR	NR	+	+	NR	+	+	NR	+	6
Zhang 2020 (19)	+	NR	+	+	+	NR	+	+	NR	+	7
Zhang 2019 (20)	+	NR	NR	+	+	NR	+	+	NR	+	6
Yin 2019 (21)	+	NR	NR	+	+	NR	+	+	+	+	7
Yang 2020 (22)	+	NR	+	+	+	+	+	+	+	+	9
Xie 2021 (23)	+	NR	NR	+	+	NR	+	+	NR	+	4
Wang 2021 (24)	+	NR	+	+	+	–	+	+	+	+	8
Sun 2021 (25)	+	NR	+	+	+	NR	+	+	NR	+	7
Sun 2020 (26)	+	NR	+	+	+	+	+	+	+	+	9
Shi 2018 (27)	+	NR	NR	+	+	NR	+	+	NR	+	6
Li 2021 (28)	+	NR	NR	+	+	NR	+	+	+	+	7
Li 2019 (29)	+	NR	+	+	+	NR	+	NR	+	+	7
Li 2019 (30)	+	NR	+	NR	+	NR	+	+	NR	+	6
Li 2021 (31)	+	NR	NR	NR	+	NR	+	NR	NR	+	4
Li 2019 (32)	+	NR	NR	+	+	NR	+	+	+	+	7
Li 2021 (33)	+	NR	+	NR	+	+	+	+	NR	+	7
Hu 2019 (34)	+	NR	+	+	+	NR	+	NR	+	+	7
Dai 2021 (35)	+	NR	+	NR	+	NR	+	+	NR	+	6
Chen 2021 (36)	+	NR	+	NR	+	NR	+	+	NR	+	6
Jia 2021 (37)	+	NR	NR	+	+	NR	+	NR	+	NR	5
Qian 2021 (38)	+	NR	+	+	+	NR	+	+	+	NR	7
Guan 2021 (39)	+	NR	NR	NR	+	NR	+	+	NR	NR	4
Xia 2020 (40)	+	NR	+	+	+	NR	+	+	NR	NR	6
Xu 2020 (41)	+	NR	+	+	+	NR	+	NR	NR	NR	5
Zhu 2020 (42)	+	NR	+	+	+	NR	+	+	NR	NR	6
Yao 2020 (43)	+	NR	NR	NR	+	NR	+	+	NR	+	4
Wang 2019 (44)	+	NR	+	+	+	+	+	+	+	NR	8
Meng 2018 (45)	+	+	NR	+	+	+	+	+	NR	NR	7
Zheng 2021 (46)	+	NR	+	+	+	NR	+	+	+	NR	7
Huang 2020 (47)	+	NR	NR	NR	+	NR	+	+	NR	NR	3
Zhou 2020 (48)	+	NR	+	+	+	+	+	+	NR	NR	7
Yang 2017 (49)	+	NR	NR	+	+	+	+	NR	+	+	7
Feng 2019 (50)	+	+	+	+	+	NR	+	+	+	+	9
Zhao 2019 (51)	+	NR	+	NR	+	NR	+	+	NR	+	6

1, peer-reviewed publication; 2, statements describing control of temperature; 3, randomization to treatment group; 4, allocation concealment; 5 blinded assessment of outcome; 6, avoidance of anesthetics with known notable intrinsic neuroprotective properties; 7, use of animals with relevant comorbidities; 8, sample size calculation; 9, compliance with animal welfare regulations; 10, declared any potential conflict of interest +: The article confirms the indicator and gets a point. -: This article does not meet this indicator and this indicator is not scored. NR: No description of the indicator in the article.

## Discussion

According to relevant reports, cancer has long remained the leading cause of death worldwide (53), such as breast cancer (54), lung cancer (55), liver cancer (56), and colon cancer (57). Traditional clinical diagnosis and treatment measures can no longer meet the needs of precise targeted therapy and even individualized treatment for these cancers (12). Although the commonly used measures PET, CT, MRI and US have made great achievements in tumor detection, the inevitable problems of low resolution, shallow imaging depth and certain radiation to organisms make people have to explore more excellent precise diagnosis strategies. Therefore, the optical imaging technology with high sensitivity and biological safety has been developed rapidly. Compared with visible light (400-700 nm) and NIR-I imaging, NIR-II technique with higher penetration depth and lower scattering has been generally accepted by researchers (10). It has been pronounced that the penetration depth of NIR-II imaging can reach more than 7 mm (58), contributing to identify and distinguish tiny tumors that cannot be discerned by traditional imaging strategies (59).

Conventional tumor diagnostic equipment is difficult to be integrated into the operating room and unable to provide real-time feedback of anatomical site information for aggressive surgery. Inconceivably, NIR-II guided tumor excision has conquered this conundrum in laboratory animals because of its wide imaging field and real-time feedback of tumor sites (60–62). Stupendously, the distinctive photothermal properties of some NIR-II probes enabled to convert light energy into heat under NIR laser irradiation, thereby inducing apoptosis of tumor cells. Robust evidence has manifested that NIR-II-guided PTT has unique advantages in accurate diagnosis and non-invasive treatment of tumors in comparation with chemotherapy and surgical resection (63).

**Figure 5:**
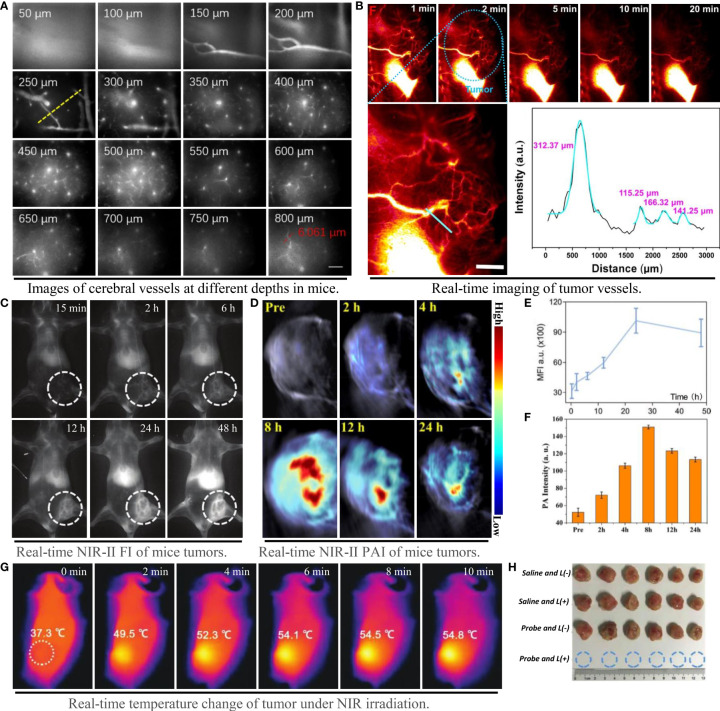
Imaging capability of NIR-II probe on blood vessels and tumors, and effect of photothermal therapy. **(A)** Probe IR-820 imaging of cerebral blood vessels in mice at different tissue depths (scale bar: 100 μm) (50); **(B)** High resolution imaging of tumor vessels at magnification and gaussian fitting curve of vessel cross section (scale bar: 2 mm) (29); **(C–E)** NIR-II fluorescence imaging and fluorescence intensity of mouse tumors at different time points (31); **(D–F)** NIR-II photoacoustic imaging and photoacoustic intensity of mouse tumors at different time points (26); **(G)** Tumor temperature changes at different time points under NIR laser irradiation (18); **(H)** Tumor photos and volume changes after photothermal treatment under different conditions, from top to bottom: 1) saline; 2) saline + laser; 3) TSSI NPs; 4) TSSI NPs + laser (18).

Qualified NIR probes with good biocompatibility and excellent fluorescence performance can be divided into two categories: organic materials and inorganic materials. The former includes molecule fuels, aggregation induced emission molecules and nano-polymers, while the latter comprises quantum dots, single wall nanotubes and rare earth nanoparticles (64). The luminance of the contrast agent in the NIR-II region is proportional to the product of QY and ϵ at the excitation wavelength (22). Inorganic probes generally have high QY and photobleaching properties, but most of them have potential biological toxicity with the presence of toxic metal ions (65, 66). Although the biosafety of most organic fluorophores is better than that of inorganic materials, the short tumor residence time, low water solubility and QY prevent their widespread use. The lack of clinically approved NIR-II probes with high luminance and biosafety, and the inability to construct new PTA on this basis, is the main bottleneck that limit the development of PTT (67). However, the design philosophy based on structural modification and enhancement have made some progress in both safety and fluorescence properties of organic small molecules (68). A small molecule organic probe IR-820 (50) with high QY (ϵ=30 L/g·cm, QY=2.52%), reportedly showed unrivalled fluorescence brightness and could accurately image cerebral vessels at depth of 800 μm (**Figure 5A**). The semiconductor polymer L1057 NPs (ϵ=18 L/g·cm, QY=1.25%) can visualize 1.9 μm blood vessels under high power microscope (22). In addition, the probe NaLuF_4_ NR_S_@PDA (29) with exceptional fluorescence emission and QY, can even illuminate the tiny blood vessels connected to the tumor with extreme clarity (**Figure 5B**).

The accuracy of NIR-II system in tumor diagnosis is determined by specific probes for tumor recognition. The massive accumulation of NIR-II probes at the tumor cell proclaimed intensive signal than that of surrounding tissue, which is conducive to the effective identification of tumors. Acknowledgedly, the local interactions between tumor cells and their adjacent tissues make tumor microenvironment (TME) slight acidity and overexpression of H_2_O_2_ (69). In the circumstances, Ag_2_S-GOx@BHS NYs, a TME specific activated probe, accumulated and ignited heterogeneous tumor (15). As a crucial organelle of ion metabolism, mitochondria are also the focus of NIR-II probe design for targeted tumor imaging. H4-PEG-PT was successfully designed to selectively capture and track mitochondria of osteosarcoma cells with a subcellular resolution of 1 nM (48). In addition, the enhanced permeability and retention effect also provides an opportunity for NIR-II probe to light the tumor through aggregated luminescence, such as PCTA-BMA-OXA (42) and SPIO@NC (39).

The selection of laser light source, the time of laser irradiation and the period of treatment are all considered as potential factors during PTT. In order to reduce the injury of laser to skin, the photothermal treatment generally chooses the laser with lower than maximum permissible exposure (33) as the irradiation light (e.g., 0.3 W/cm^2^ at 808 nm and 1 W/cm^2^ at 1064 nm). In the analysis of this paper, it was found that the start time of laser irradiation was generally determined by the emergence time of the maximum fluorescence or photoacoustic signal of the probe *in vivo*. The photothermal transformation of the probe could be achieved to the maximum extent when the accumulation quantity of the probe reached the utmost in the tumor tissue (**Figures 5C–F**). As a PTA converting light energy into heat to kill tumor cells, no applicable rules declared the period of PTT in the included studies, which should be further investigated (70). When the NIR-II probe is excited to make the local tissue temperature of tumor exceed 43°C, it will produce irreversible effect of killing tumor cells (7). At present, other than affecting mitochondrial metabolism, there is no definite conclusion on the specific mechanism of PTT. Evidence has suggested that PTT can activate TRPV1 ion channel in tumor cells and heighten the intracellular Ca^2+^ level, causing mitochondrial dysfunction and apoptosis (71). Thereupon, dysfunctional mitochondria can further whittle the body’s resistance to thermal damage by stimulating the expression of heat shock proteins (72). Promisingly, the especially designed PTA can generally kill tumors by heating up to more than 43°C in a quick time after sensing NIR laser at the tumor site (**Figure 5G**). Most NIR-II probes with properly photothermal properties such as TSSI NPs (18), can even achieve tumor ablation completely (**Figure 5H**).

It should be noticed that PTT is often distinguished from photodynamic therapy (PDT) due to the fact it does not directly depend on cellular oxygen tension. PDT principally relies on photosensitizers to generate reactive oxygen species under light irradiation to induce tumor cell death (73). In view of the divergent mechanisms of these two methods, PTT may be more suitable for the treatment of some hypoxic tumor, while PDT is inclined to most effective for hyperoxia tumor (74). Moreover, the intensity of the NIR laser attenuates extensively with the depth of penetration, resulting in a sudden drop in available heat energy for deep tumor mass. This limitation makes PPT more suitable for subcutaneous or other tumors closer to the body surface. In order to amplify the applicability of PTT, the combination of photothermal treatment and PDT, immunotherapy, chemotherapy and gene therapy has been established and achieved remarkable results (75–78).

In this paper, we determined some evidence about the efficacy and safety of PTT for cancer remedy using a meta-analysis method. Whereas there are still some tricky problems need to be solved. First of all, the small sample size and some ineluctable low-quality preclinical researches have discounted the consequences of the meta-analysis to some extent. Secondly, the data available for forest plot analysis is too scarce, so we solely used the cases of tumor complete ablation and suppression for correlation analysis. The influence of treatment conditions such as NIR laser intensity and irradiation time needs to be analyzed with more complete research data. Although NIR-II guided PTT is a promising strategy for cancer treatment, there are still many bottlenecks urgently need to be addressed before its clinical application. Sanguinely, we hold that the continuous development of NIR-II imaging technology and nanotechnology will eventually overcome the existing issues and bring more extensive application of PTT in the future.

## Conclusions

Based on a meta-analysis of preclinical studies, this paper demonstrated that PTT guided by NIR-II imaging has terrific efficacy and high safety in cancer treatment. Evidence from multiple preclinical data indicated that PTT will be a promising strategy for clinical multi-cancer therapy. Nevertheless, the development of novel NIR-II probes that are more suitable for clinical use is still the core issue of PTT therapy. Meanwhile, the potential multiorgan toxicity of probes also requires extensive preclinical and clinical evaluation.

## Author contributions

The main conception and design of this paper: FF, YaZ, XM, and XW. Data extraction and sorting: FF, YaZ, YZe, YZh and SZ. Article drafting: FF and YH. Modification and optimization: FF and XW. All authors contributed to the article and approved the submitted version.

## Funding

This study was supported by the National Natural Science Foundation of China (82104533 and 81973569), the Science & Technology Department of Sichuan Province (2021YJ0175), and the China Postdoctoral Science Foundation (2020M683273).

## Conflict of interest

The authors declare that the research was conducted in the absence of any commercial or financial relationships that could be construed as a potential conflict of interest.

## Publisher’s note

All claims expressed in this article are solely those of the authors and do not necessarily represent those of their affiliated organizations, or those of the publisher, the editors and the reviewers. Any product that may be evaluated in this article, or claim that may be made by its manufacturer, is not guaranteed or endorsed by the publisher.
